# FIGO Stage IV and Age Over 55 Years as Prognostic Predicators in Patients With Metastatic Malignant Struma Ovarii

**DOI:** 10.3389/fonc.2020.584917

**Published:** 2020-09-29

**Authors:** Sijian Li, Tengyu Yang, Xiaoyan Li, Limeng Zhang, Honghui Shi, Ninghai Cheng, Jinghe Lang

**Affiliations:** ^1^Department of Obstetrics and Gynecology, Peking Union Medical College Hospital, Chinese Academy of Medical Sciences, Peking Union Medical College, Beijing, China; ^2^Department of Otolaryngology, Peking Union Medical College Hospital, Chinese Academy of Medical Sciences, Peking Union Medical College, Beijing, China

**Keywords:** malignant struma ovarii, metastatic, treatment, prognosis, risk factors

## Abstract

**Introduction:** Metastatic malignant struma ovarii (MSO) is an extremely rare disease that lacks treatment consensus and accurate prognosis. The objective of this study was to present the clinical, pathological, and treatment characteristics of metastatic MSO, while also investigate the overall survival (OS) rate and factors affecting prognosis in this population.

**Materials and Methods:** A total of 79 cases of metastatic MSO were reviewed, including four cases of metastatic MSO from our hospital and 75 cases selected from the literature. Logistic regression was used to identify potential factors affecting disease free survival (DFS). The Kaplan-Meier method and log-rank test were used to determine OS; further Cox regression was used to evaluate factors affecting OS.

**Results:** The mean age of all the patients at diagnosis was 43.8 years. The most common metastatic sites were peritoneum, bone, liver, omentum and lung in descending order. Only two patients (2.6%) coexisted with local primary thyroid cancer. Follicular carcinoma (41.8%) as the most prevalent subtype, followed by papillary carcinoma, follicular variant of papillary carcinoma, and mixed follicular-papillary carcinoma. 36.7% of the patients received conservative surgery, 43.0% of them underwent aggressive surgery, and 15.2% of them did not receive any surgery. 74.7% of patients who received adjuvant therapy underwent radioiodine therapy (RAI). Logistics regression revealed that FIGO stage IV was the only prognostic factor in predicting DFS (*P* = 0.002; Odds Ratio [OR] 5.333; 95% confidence interval [CI]: 1.839–15.471). Only seven deaths occurred. The OS rates at 5, 10, 15 years were 89.3, 82.4, 65.9%, respectively. Multivariate analysis showed age over 55 years (*P* = 0.006; OR 9.362; 95%CI: 1.895–46.246) was the only risk factor for OS.

**Conclusion:** Patients with metastatic MSO have an excellent disease-specific OS rate, FIGO stage IV and age over 55 years were two factors affecting disease prognosis. Conservative surgery with residual ablation by RAI after total thyroidectomy should be preferred since the benefits of aggressive surgery are uncertain.

## Introduction

Ovarian teratomas are the most common germ cell tumors and represent ~20% of all ovarian tumors (1); these tumors contain tissue from the three germ layers, typically including hair, skin, bone, and thyroid tissue. Struma ovarii is a rare form of ovarian teratomas, are composed of more than 50% thyroid tissue (2), and account for about 2% to 5% of ovarian teratomas and 0.5 to 1% of all ovarian tumors (3–5). Between 5 and 10% of struma ovarii cases are defined as MSO by the World Health Organization (WHO) and are histologically identified to differentiate from thyroid carcinomas (6). Currently, there are fewer than 200 cases of MSO reported in medical literature (7), and MSO with metastasis has been reported in 5–20% of all cases (3, 8–10). Due to its rarity, the standard management of MSO remains controversial.

Previous publications advise aggressive treatment in combination with a total thyroidectomy, postoperative radioiodine therapy (RAI), and thyroid hormone suppressive therapy, regardless of the presence or absence of metastasis at diagnosis (11–14). In these studies, conservative surgery was optional in childbearing patients, while they recommended comprehensive staging surgery including total abdominal hysterectomy, bilateral salpingo-oophorectomy, pelvic washings, and pelvic lymph node sampling if fertility preservation was not a desired option (14, 15). Recently, two of the largest studies, reported by Goffredo et al. (8) and Robboy et al. (16), revealed an excellent disease-specific survival rate of MSO, regardless of disease management strategy employed. Furthermore, a study done by McGill et al. recommended a total thyroidectomy in conjunction with RAI therapy in the case of metastatic struma ovarii (17), but they did not mentioned which exact surgical approach should be applied in this population. Another study from Marti et al. showed that extensive pelvic surgery and a prophylactic total thyroidectomy might be beneficial in patients with metastatic MSO (18).

While previous research has been conducted in the field, none of these studies were restricted to metastatic MSO; neither an optimal disease management strategy nor long-term survival rates for this population were well-defined and much remains uncertain. It was undetermined whether aggressive surgery should be implemented in these patients rather than a more conservative surgery, and the role and efficacy of various postoperative adjuvant therapies needed to be evaluated thoroughly.

To describe the clinical, pathological, and treatment characteristics among patients diagnosed with metastatic MSO, investigate disease-free survival (DFS) and overall survival (OS) rate, and determine factors affecting OS and DFS in this population, we selected four cases of metastatic MSO and comprehensively reviewed another 75 cases documented in literature from MEDLINE in the last 80 years. Dilemmas in treatment for pregnant women with metastatic MSO were also discussed.

## Materials and Methods

The retrospective analysis of four cases in our hospital was approved by the Ethic Committee of Peking Union Medical College Hospital (reference number: S-K1198). We identified four cases of metastatic MSO between 1990 and 2020. Demographic data and clinical, pathological, and treatment characteristics were obtained and confirmed by treating physicians. A systematic literature review on metastatic MSO cases from 1940 to 2020 was performed using PubMed (http://www.pubmed.gov), using the following keywords to select for studies: “metastatic malignant struma ovarii;” “malignant struma ovarii;” “malignant ovarian teratoma;” “struma ovarii;” “metastatic thyroid carcinoma arising in struma ovarii.” Patients with MSO lacking extra-ovarian spread, metastatic MSO found by autopsy, benign metastatic struma ovarii (histologically benign but clinically malignant), or lack of demographic data, pathological information, or treatment details were excluded. After the elimination process, 75 cases of metastatic MSO were selected to be reviewed (The detailed inclusion process can be found in Supplementary Figure 1). A database was generated including demographic features, survival outcomes, and clinical, pathological and treatment characteristics from these 75 cases, as well as the four cases in our hospital. Clinical, pathological, and treatment characteristics were analyzed to identify independent variables that might predict disease prognosis, including age at diagnosis [<55, ≥55years, the age cut-point was selected as 55 years in accordance with the American Joint Committee on Cancer (AJCC) staging system for well-differentiated thyroid cancers (19)], time till metastasis (metastasis at initial presentation or not), pathological subtypes (follicular carcinoma or not), FIGO stage (20) (stage II–III or IV), surgical intervention (no surgery, conservative surgery or aggressive surgery), and adjuvant therapy (with or without RAI). In this study, conservative refers to ovarian cystectomy with or without metastases resection, simple metastases resection, unilateral salpingo-oophorectomy (USO) with or without metastases resection, bilateral salpingo-oophorectomy (BSO) while aggressive surgery includes total abdominal hysterectomy and BSO (TAH+BSO) and debulking surgery (TAH+BSO, with omentectomy and/or appendectomy, lymph nodes resection, etc.), concerning the scope of surgery as well as the impact on females' reproductive system.

## Statistical Analysis

Continuous variables are described by means ± standard deviation (range) if they were normally distributed or as medians and interquartile ranges (IQRs) if they were abnormally distributed. Discrete variables are expressed as counts (percentage). Variables were compared between three outcomes (no evidence of disease, alive with disease, or died of the disease) using univariate analysis. Continuous variables were compared using a Student's *t*-test or the Mann–Whitney *U*-test, depending on their distribution. Categorical variables were compared by the chi-squared test. Variables in the univariate analysis with *p* < 0.2 were selected for potential inclusion in the multivariate logistic regression. Backward, stepwise logistic regression was carried out to determine potential prognostic factors. Odds ratios (OR) with 95% confidence intervals (CI) and *p*-values were calculated. Overall survival curves were established using the Kaplan-Meier method, and survival rates between subgroups were compared by the log-rank test. Univariate analysis was performed to assess clinical prognostic factors for OS. Factors with *p* < 0.2 were subjected to multivariate analysis using the Cox proportional hazards model to identify independent prognostic factors for OS. A two-tailed *p* < 0.05 was considered significant. Statistical analysis was conducted using SPSS (Version 21.0; SPSS Inc., Chicago, IL, USA) or GraphPad prism (Version 8.0) software.

## Results

### Four Case Series

#### Demographic Data and Clinical Characteristics

**Figure 1:**
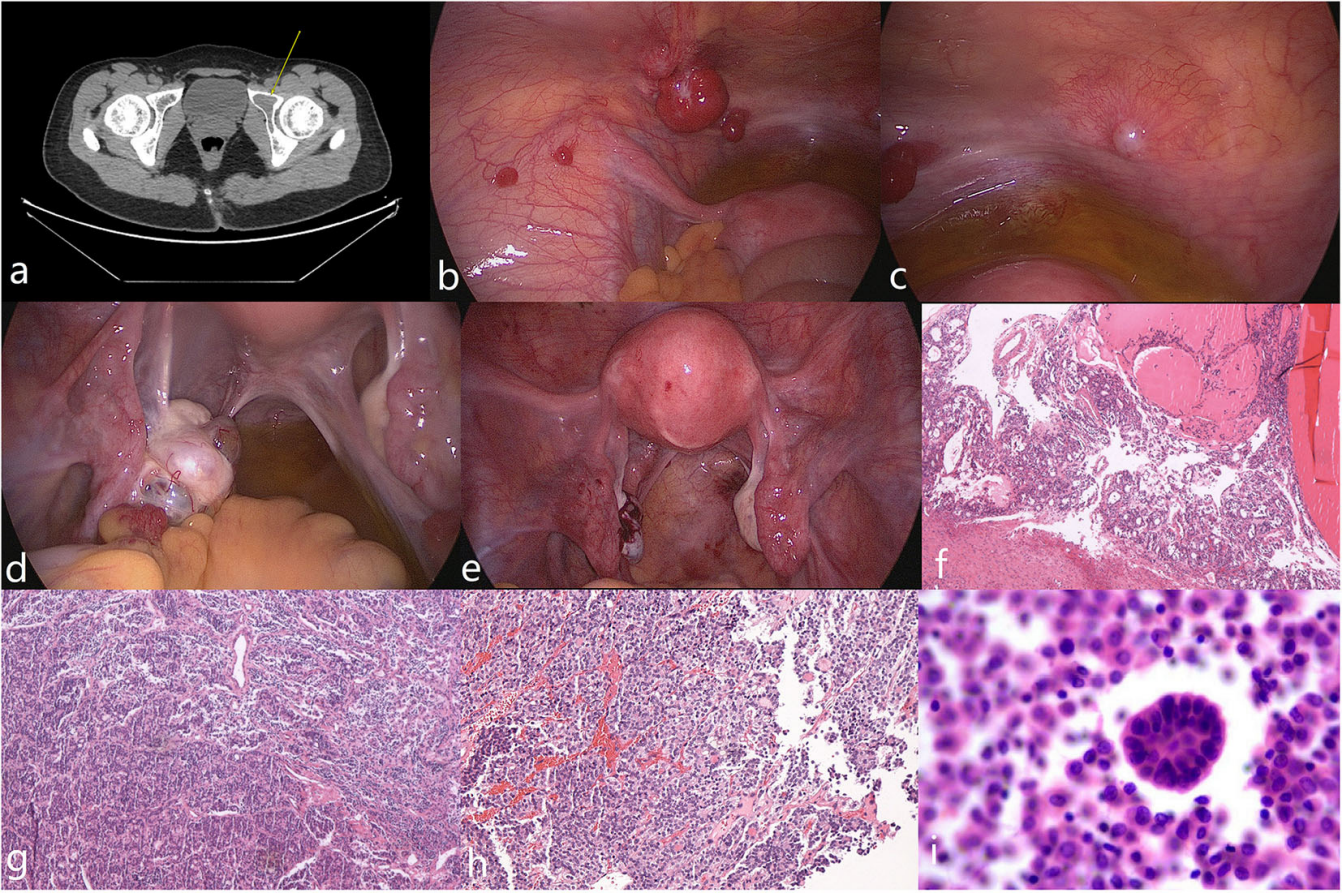
Imaging, intraoperative, and pathological photos of typical appearance of MSO metastasized to peritoneum (case 3). **(a)** Pelvic CT showed lesion on left acetabulum (arrow). **(b,c)** Multiple smooth pink nodules distributed spread on peritoneum with diameters that varied from 5 mm to 2 cm were noted. Ascites was mild with a clarified faint yellow color. **(d)** The left ovary was enlarged; a pink nodule on its surface with a 1-cm in diameter was noted. **(e)** Postoperative appearance of the pelvic cavity. **(f–h)** Pathological examinations of the left ovary, peritoneal nodules and left acetabular lesion showed features of MSO. **(i)** Malignant tumor cells were found in peritoneal washing.

The mean age of four patients at the time of diagnosis of metastatic MSO was 36 (range 33–39) years old. Their initial finding was a pelvic mass, which was identified by imaging. The mean time interval from the initial diagnosis of struma ovarii (benign or malignant) to metastatic MSO was 5.5 (range 2–9) years. Thyroglobulin (TG) was elevated in three patients, but none presented dysthyroid. The peritoneum and lungs were the most common sites of metastasis (2 of 4), followed by the omentum, bone, uterus, and vaginal residue (1 of 4, respectively) (Table 1 and Figure 1).

**Table 1:** Demographic and clinical characteristics of the four patients.

**No**.	**Age (y)**	**Initial surgery and pathology**	**CA125 elevated**	**TG elevated**	**Metastases site; pathology**	**Surgery at metastases**	**Adjuvant therapy**	**Results of follow-up**
1	39	USO(LSO) at 9 y ago; MSO	N	Y	Lung; FTC	N	TT, RAI^*^4	AWD at 10 y (WBS negative, elevated TG, 16 ng/ml)
2	38	Ovarian cystectomy at 6 y ago; benign SO	N	Y	Uterus, omentum, liver, lung; FTC	Debulking Surgery (TAH + BSO + omentectomy + pelvic lymph nodes dissection)	TT, RAI^*^4	AWD at 5 y (progress slowly on CT, elevated TG, 226 ng/ml)
3	33	Ovarian cystectomy at 5 y ago; atypical benign SO	Y	N	Peritoneum, left acetabulum; DTC	Left ovarian cystectomy, metastases resection, bone biopsy	N	AWD at 15 m (CA 125 was normal after surgery; lack of postoperative PET/iodine scan due to pregnancy)
4	34	USO(LSO) at 2 y ago; MSO	Y	Y	Peritoneum, vaginal residue; DTC	Debulking surgery (sTAH + RSO + omentectomy + appendectomy + metastases resection)	Chemotherapy (12 cycle)	AWD at 61 m (elevated TG, > 1,000 ng/ml; CA 125 was normal after chemotherapy)

*SO, struma ovarii; MSO, malignant struma ovarii; FTC, follicular thyroid carcinoma; (D)TC, (differentiated) thyroid carcinoma; USO, unilateral salpingo-oophorectomy; LSO, left salpingo-oophorectomy; BSO, bilateral salpingo-oophorectomy; TAH/sTAH, total/subtotal abdominal hysterectomy; TT, total thyroidectomy; RAI, radioiodine therapy; WBS, whole body scan; NED, no evidence of disease; AWD, alive with disease; DOD, die of the disease*.

* “RAI*4” refers to four cycles of RAI therapy; the chemotherapy protocol of patient 4 included 6 cycles of Taxol + carboplatin, followed by VP 16 + carboplatin for 2 cycles, VP 16 + cisplatin for 2 cycles, Taxol + ifosfamide (IFO) for one cycle and gemcitabine + oxaliplatin for one cycle.

#### Surgery, Pathology, and Adjuvant Therapy

All patients underwent conservative surgical treatment at initial presentation of struma ovarii, and debulking surgery was implemented in two patients at metastasis. The third patient desired preservation of fertility and received an ovarian cystectomy in combination with metastasis resection and a bone biopsy. The last patient did not undergo surgery at metastasis. Lymph nodes were examined in one patient but the results were negative. Follicular thyroid carcinoma was the most common (2 of 4) pathological pattern, while the rest were diagnosed as unspecified differentiated thyroid carcinoma.

Concerning the adjuvant treatment, two patients received a total thyroidectomy, but no malignancy was revealed; both received RAI. One patient received 12 cycles of chemotherapy. The other two patients showed a normal thyroid on whole body PET/CT scans and thyroid ultrasounds, and therefore did not undergo thyroid surgery or RAI.

#### Result of Follow-Up

All four patients survived the disease and had a median follow-up of 52.5 months (range 15–120) after diagnosis of metastatic MSO. One patient's whole-body iodine scan was negative but her serum TG level was elevated, and the patient who received an ovarian cystectomy and metastasis resection had a successful pregnancy and delivered a healthy baby during follow-up.

### Database of 79 Cases

**Figure 2:**
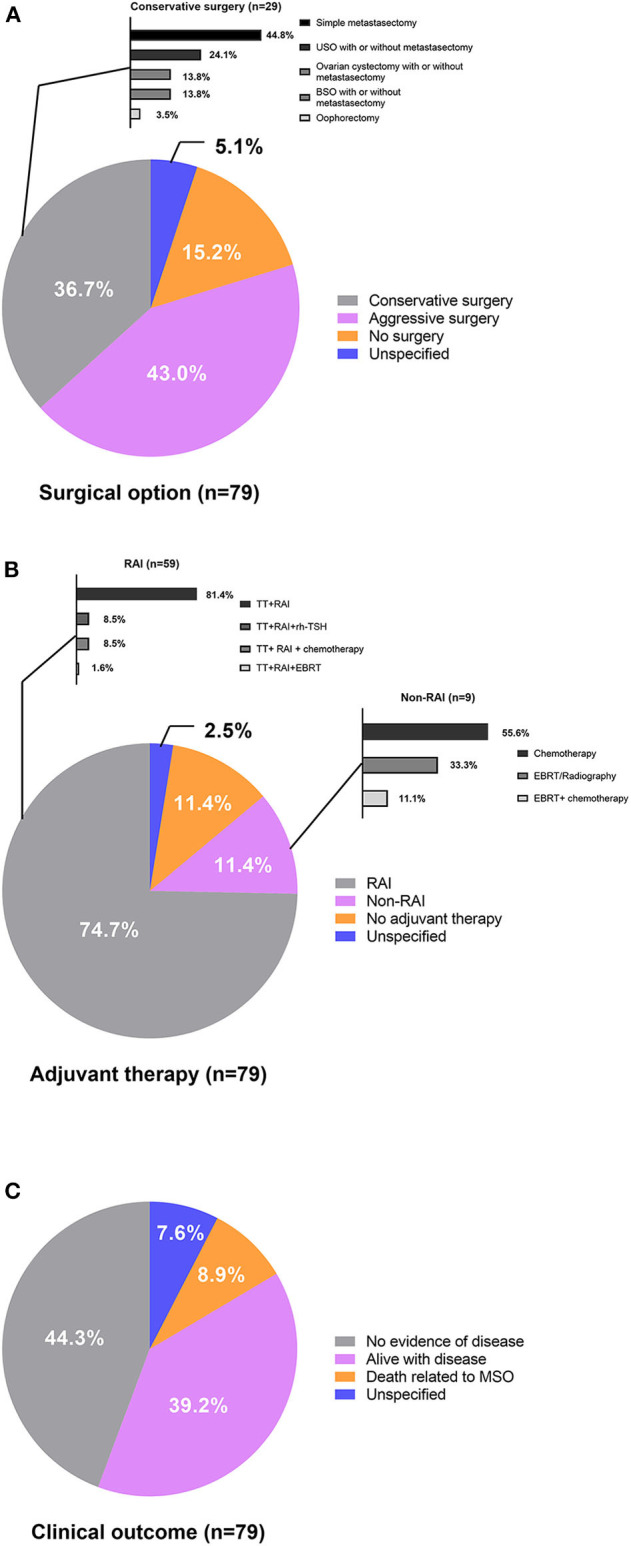
**(A)** Surgical options, **(B)** adjuvant therapy, and **(C)** clinical outcome of patients with metastatic MSO.

Seventy-nine patients with metastatic MSO were included in the retrospective analysis (details can be found in the supporting information) and clinical characteristics were recorded (Table 2 and Figure 2). The mean age at diagnosis was 43.8 years, with a median age of 42 years (range 19–76). Information on CA-125 and thyroid function was available in 18 and 53 patients, respectively. Ten cases (55.6%) had elevated levels of CA-125 and three (5.7%) presented with hyperthyroidism. Metastasis at initial presentation was reported in 37 (46.8%) cases. Metastatic MSO was diagnosed in the other 42 cases with a median delay of 4.5 years after initial presentation, ranging from 2 months to 26 years.

**Table 2:** Clinical characteristics of patients with metastatic MSO.

**Patient characteristics**	**N**	**%**	**Patient characteristics**	**N**
Age (year)	79		**Sites of metastatic lesions**	N = 79
Mean 43.8 ± 13.5			Peritoneum	36
Range 19–76 Median 42			Bone	25
**CA 125**	N = 18		Liver	20
Yes	10	55.6%	Omentum	17
No	8	47.1%	Lung	15
**Hyperthyroidism**	N = 53		Lymph nodes	10
Yes	3	5.7%	Contralateral ovary	9
**Synchronous primary thyroid carcinoma**	N = 79		Uterus	7
Yes	2	2.5%	Diaphragm	6
**Initial SO related Pathology**	N = 79		Bowel	6
Benign SO	19	24.0%	Spleen	4
Malignant SO	57	72.2%	Bladder	3
NA	3	3.8%	Mediastinum	3
**Pathology at Metastasis**	N = 79		Mesentery	2
PTC	19	24.1%	Fallopian tube	2
FVPTC	14	17.7%	Round ligament	1
FTC	33	41.8%	Skin	1
Mixed FTC+PTC	2	2.5%	Appendix	1
Unspecified	11	13.9%	Adrenal	1
Overall poor differentiated	4	5.1%	Vaginal residue	1
**Metastatic MSO at initial presentation**	N = 79		**Bone metastases**	N = 25
Yes	37	46.8%	Pelvic bone	12
No	42	53.2%	Rib	4
**Time to metastasis (year)**	N = 42		Spine	5
Mean 6.5 ± 6.2			Scapula	3
Range 0.17–26 Median 4.5			Skull	3
**Lymph node examination**	N = 14		Femur	2
Positive	5	35.7%	Unspecified	1

*SO, struma ovarii; MSO, malignant struma ovarii; PTC, papillary thyroid carcinoma; FTC, follicular thyroid carcinoma; FVPTC, follicular variant of papillary thyroid carcinoma; NA, not applicable*.

For the initial SO related pathology, 19 (24.0%) cases were diagnosed with benign SO and 57 (72.2%) with malignant SO (including cases corrected for after pathological review). The final pathology reports from metastatic resections revealed a range of thyroid carcinoma subtypes with follicular carcinoma (41.8%) as the most prevalent subtype. Other reported subtypes, in order of prevalence, were papillary carcinoma, follicular variant of papillary carcinoma, and mixed follicular-papillary carcinoma. The proportion of follicular carcinoma cases was 55.3% (21 cases) and 29.3% (12 cases) before and after 2010, respectively. Nonetheless, the percentage of follicular variant of papillary carcinoma cases was 10.5% (four cases) and 24.4% (10 cases) before and after 2010, respectively. A total of four cases were classified as poorly-differentiated thyroid carcinoma, and two patients (2.5%) coexisted with local primary thyroid cancer.

In our study, the peritoneum was the most commonly involved site, followed by bone, liver, omentum, and lungs, in descending order. Other locations, such as contralateral ovaries, lymph nodes, uterus, bowel, and diaphragm, were less common. Spleen and mediastinum occasionally would show tumor infiltration in cases of distant metastasis. In the cases of bone metastasis, the pelvic bone was the most favorable, followed by the spine; the ribs, scapula, skull, and femur can also be involved. Bladder, mesentery, round ligament, vaginal residue, appendix, adrenal glands, and skin were uncommon metastatic sites.

Conservative surgery was performed in 29 (36.7%) cases, 34 (43.0%) cases underwent aggressive surgery, and 12 (15.2%) cases did not receive tumor resections. Surgery was unspecified in four cases (5.1%). Most reported cases (86.1%) were administrated adjuvant therapy, including RAI after total thyroidectomy, radiation therapy, or external beam radiation treatment (EBRT) and chemotherapy. Total thyroidectomy followed by RAI was the most predominantly used adjuvant treatment strategy and accounted for 74.7% of the total patients who received adjuvant therapy. Five cases treated with RAI therapy after total thyroidectomy were assisted by recombinant human TSH (rh-TSH). Chemotherapy, radiation therapy/EBRT, or a thyroidectomy followed by RAI were also treatments adopted, but the regimens varied among patients and institutions. Serum thyroglobulin levels were monitored after total thyroidectomies to reflect tumor burdens for most cases.

Follow-up data were available for 73 cases and there was a wide range in the length of follow-up among the reported cases. Thirty-five (44.3%) cases achieved no evidence of disease (NED), 31 (39.2%) cases were alive with disease (AWD), and 7 (8.9%) cases died of the disease (DOD) at the final follow-up. The rest six cases (7.6%) did not report follow-up information. The results of the univariate and multivariate analyses identified risk factors associated with AWD/DOD (Supplementary Table 2), and univariate analysis revealed that a more advanced stage of disease was associated with AWD/DOD. More aggressive surgical interventions had a possibility for association with better prognoses. Stage IV disease (*p* = 0.002; OR: 5.333; 95%CI: 1.839–15.471) was identified as the only risk factor for AWD/DOD in further multivariate logistic regression.

**Figure 3:**
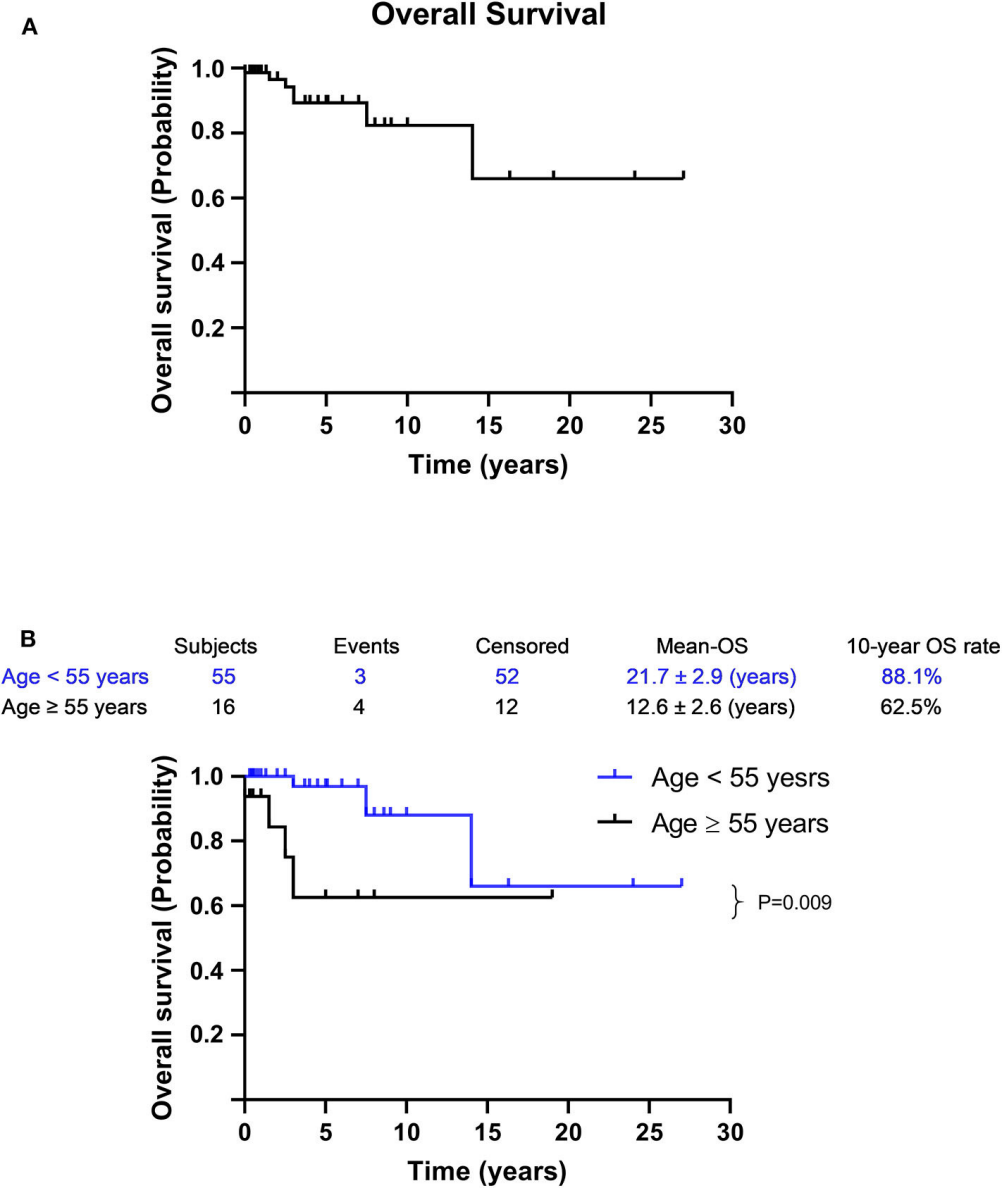
Survival curves for metastatic MSO. **(A)** The OS for all patients with metastatic MSO. **(B)** Age < 55 vs. age ≥ 55 years. It was analyzed by Kaplan–Meier (log-rank) test. Percentages of survival patients are shown at indicated time points. Censored patients are marked with “**∣**” in the graph.

Only seven deaths occurred in this cohort. The 5, 10, and 15-years OS rates were 89.3, 82.4, 65.9%, respectively (Figure 3). The mean OS was 20.9 years (95%CI: 16.1–25.6), and factors that may affect survival outcomes were summarized (Supplementary Table 3). Univariate analysis showed that age (<55 y vs. ≥55 y; *p* = 0.009) was significantly associated with disease prognosis, while a more advanced FIGO stage disease was likely to predict a poor prognosis. Age and FIGO stage were used for further multivariable analysis, where an age >55 years (*p* = 0.006; OR: 9.362; 95%CI: 1.895–46.246) was statistically significant.

## Discussion

Our study presents the largest cohort of patients diagnosed with metastatic MSO and reveals that MSO prognosis is still satisfactory even after metastasis, regardless of the treatment strategies used. However, age over 55 years predicts a lower overall survival rate and a FIGO stage IV is directly related to a decreased probability of disease-free survival. This article may pose to shed new light on the optimal management of metastasis MSO.

Nearly half of the patients in our study initially presented with metastases, and for those who subsequently developed metastases, the time interval from initial struma ovarii related pathology to the identification of metastatic MSO varied from 2 months to 26 years. This made it challenging to determine a timely diagnosis of metastatic MSO and emphasized the significance of consistent follow-up, even in patients diagnosed with benign struma ovarii. Of the metastatic sites of MSO, the peritoneum was the most common involved (45.6%), followed by bone, liver, omentum, and lungs, in descending order. Surprisingly, bone was the second most common site and only two cases (2.5%) showed to be metastasizing to the mesentery, which was not compatible with a previous study (17).

In the present study, we noted hyperthyroidism in 5.7% of patients, which is compatible with a previous study that found struma ovarii is usually non-functional, particularly in malignant conditions (21). Goffredo et al. found that 8.8% of patients with MSO had a concomitant or subsequent diagnosis of thyroid cancer; two thirds of which experienced extended cancer outside the thyroid gland (8). Alternatively, only two patients (2.5%) from our data coexisted with primary thyroid carcinoma confined to the thyroid gland. The rate in our study was much lower, but it was still higher than that of the general population (22). This increased incidence may be due to rigorous thyroid surveillance, such as imaging or thyroidectomies, in this special population. The synchronous arising of multifocal thyroid-type tumors in separate sites may also be explained by the shared molecular susceptibility to neoplastic transformation during embryogenesis, which is triggered by a carcinogen-damaged field, or “field cancerization” (12). All this evidence suggests to us that more attention is needed on synchronous thyroid disorders in patients suffering from MSO.

Unlike primary thyroid carcinoma, the most common histologic subtype of metastatic MSO was follicular carcinoma, followed by papillary carcinoma, follicular variant of papillary carcinoma, and mixed follicular-papillary carcinoma. Seventy-two percent of the patients with a follicular carcinoma subtype had a FIGO stage IV disease, which mimics the propensity for hematogenous spread of primary follicular thyroid carcinoma (23). However, the prevalence of follicular carcinoma cases decreased in recent years. Since the definition of follicular variant of papillary carcinoma was first introduced in 1960's (24) and our cases go back to 1940's, some follicular variant of papillary carcinoma cases might be mistakenly diagnosed as follicular carcinoma, which biased the proportion of pathologic subtypes.

This is the first study that shows that patients with metastatic MSO have an excellent OS rate; previous studies on long-term survival in patients with MSO were not strictly confined to women with metastatic disease. Robboy et al. in 2009 stated that the OS rate for patients with MSO was 89% at 10 years and 84% at 25 years (16). Similarly, a study including 68 cases of MSO published by Goffredo et al. in 2015 showed that the OS rate at 5, 10, and 20 years were 96.7, 94.3, and 84.9%, respectively (8). Current findings in our study extend these conclusions. Nevertheless, compared with other histological patterns of ovarian carcinoma, patients with MSO have excellent disease-specific survival rates, regardless of whether metastasis occurred or any of the various treatment strategies were employed. Epithelial ovarian carcinoma patients with an advanced stage had a poor prognosis even after treatment progressed (25, 26). According to the FIGO staging (20), most cases in our series were stage III or IV, with the latter being most predominant: 52 (65.8%) cases were stage IV. In our study, crude overall survival in patients with metastatic MSO approached an excellent rate of 82.4% at 10 years despite mostly (76, 96.2%) having a stage III/IV disease. These findings remind us to treat MSO as a unique ovarian carcinoma that bears resemblance to primary thyroid carcinoma, and that the routine guidelines of ovarian carcinoma may not be suitable for this population. However, as an extrathyroidal manifestation of thyroid cancer, MSO was almost always considered as gynecological tumor at initial diagnosis and TNM staging was not applicable for its assessment. It might be difficult to compare the prognosis of MSO and primary thyroid cancer of the same stage.

Previously, most authors argued in favor of aggressive treatment in the case of MSO, including local surgery followed by total thyroidectomy and RAI, regardless of the presence or absence of distant metastasis (11–14). In our study, some patients underwent extensive or radical procedures to achieve minimal residual disease. There were also those who only underwent conservative surgeries and received adjuvant therapy in lieu of further aggressive surgeries when diagnosed with metastatic MSO. Some even declined all surgery and were merely treated with adjuvant therapy. We found that there was no statistically significant difference in clinical outcomes for patients treated with different approaches of surgical intervention at metastasis (*p*-value for different surgical approaches was 0.161 in DFS and 0.639 in OS), which may be attributed to the indolent natural history of MSO and the positive response to adjuvant therapy, especially in the case of RAI. We believe that, compared to mere adjuvant therapies, conservative surgery to decrease tumor burden and remove sub-centimeter lesions that benefit minimally from RAI would be a better choice. More aggressive surgery, especially comprehensive debulking surgery may impact the long-term quality of life and fertility of affected women more evidently than conservative surgery. With highly effective adjuvant therapy, conservative surgery may be the optimal choice for balancing therapeutic effects and quality of life.

Remnant ablation by RAI is a well-established treatment method for post-surgically differentiated thyroid carcinoma and can provide beneficial treatment for iodine-avid advanced disease (27). Current studies on RAI in MSO show similar results (14, 16, 17). DeSimone et al. reviewed 24 cases in 2003 and all eight recurrences were found in conservatively managed patients. RAI for recurrent disease provided an initial complete response in seven women (14). These researchers argued that treatment using a thyroidectomy and RAI should be considered as the first line of management for MSO. Even though there is no current guideline for the treatment of MSO, the majority of patients in our study were treated by a thyroidectomy followed with RAI, which was compatible with the study published by McGill et al. in 2009 that included both benign and malignant metastatic struma ovarii (17). We noted that patients treated with RAI showed no statistically significant difference in OS (*p* = 0.411) compared to other adjuvant therapies, and this could be explained by the heterogeneous nature of the patients and sample size. Patients in our study were sporadic cases from different institutions at different times, with some of them receiving over two sessions of RAI of varying doses. Significance in between-hospital variations of RAI use (28) and dosage-related successful ablation rates (29) may increase the heterogeneity of patients. While RAI may be the only feasible method of effective treatment in cases of multiple liver, lung, or bone metastases, we also recommend that postoperative RAI is the preferred therapy for patients with residual or metastatic disease. Other adjuvant therapies, such as chemotherapy and radiotherapy, were less commonly used in metastatic MSO. The number of patients treated with radiotherapy alone was too small to thoroughly evaluate treatment efficacy, while chemotherapy has been shown to be inefficient in disease control (30). One of our patients (case 4) confirmed that disease progressed even after 12 cycles of chemotherapy. Adjuvant radiotherapy and chemotherapy should be considered for RAI-refractory metastatic MSO.

Risk stratification for MSO has been proposed to guide treatment. Yassa et al. (3) suggested that patients with tumors > 2 cm, disease outside the ovaries, or aggressive histological features should be considered for postoperative RAI therapy, similar to primary thyroid carcinoma. However, measuring tumor size in a situation where cancer and teratomatous components blend together would be extremely difficult. In addition, Janszen et al. (31) found that applying the stratification criteria of primary thyroid carcinoma to MSO would result for most patients in total thyroidectomy followed by RAI. Moreover, current risk stratifications lack evidence from large sample sizes and seldom include many patients with extraovarian metastasis. In this study, we found that FIGO stage was the independent factor in predicting disease condition following treatment. This is not surprising, given that an earlier stage may have less of a tumor burden and more favorable histology. Differentiated thyroid carcinoma is only cancer to include age as part of the AJCC staging system (19), and older age has been reported as an adverse prognostic factor in thyroid cancer (23, 32). We also found that age over 55 years was an independent factor associated with mortality, with the youngest patient that died of the disease being 48 years old. It is unclear why older patient age is a strong prognostic factor for mortality in thyroid cancer. Shah et al. indicated that an excellent response to RAI was less likely to be achieved in older patients (33), possibly due to age-dependent variations in the expression of the iodine transporter that affects iodine uptake (34).

It was found that 25.3% (20/79) of patients were younger than 35 years at the time of diagnosis, which is in the time period of child-bearing age. There is little literature on pregnancy or fertility preservation in MSO that has been published (35–39). Even though it is extremely rare, complications in fertility preservation and management of pregnancy in metastatic MSO cannot be neglected. Ihalagama et al. reported an uneventful pregnancy after USO, a total thyroidectomy, and RAI in a patient with non-metastatic MSO (35). Larger et al. (39) and Tokuda et al. (38) presented disease management in cases of accidental diagnosed with metastatic MSO after conception. The former case was treated with USO and RAI, while the latter was treated with a simple metastasis resection, but this case lacked follow-up information. Salvatori et al. (36) and Fabbri et al. (37) reported a 22 years old woman diagnosed with metastatic MSO successfully obtained NED after an ovarian cystectomy and metastases resections combined with a total thyroidectomy and RAI. However, after NED for 10 years, transplantation of a cryopreserved ovarian failed to improve premature ovarian failure, which might have been caused by repeated ovarian surgeries. Large scale studies indicated that RAI ablation does not decrease overall birthrate among women (40) or lead to adverse obstetric outcomes when conception occurs 6 months or more after treatment (41). However, patients with multiple RAI therapies may experience a further decrease in ovarian reserves compared to those who received a single RAI therapy, especially in females over 35 years of age (42). These researches indicate the dilemma in balancing fertility preservation and therapeutic efficacy: the paradox of prioritizing between pregnancy and treatment. An advisable treatment for women in this population who desire to preserve fertility may be conservative surgery and tumor resection combined with total thyroidectomy followed by a less aggressive postoperative RAI to facilitate a favorable prognosis.

The study has some limitations that must be disclosed. First, the retrospective nature and heterogeneity of cases from different articles potentially affects the validity of the analysis, but the rarity of the disease makes it unpractical to conduct prospective research. Second, surveillance methods varied slightly among studies, including CT; whole body radioiodine scans combined with serum thyroglobulin level may have affected true DFS. Surgical intervention also varied even in the same category, such as debulking surgery, due to procedures being done by different surgeons, which may underestimate DFS obtained from different surgical approaches. Further studies, including case reports, are warranted to optimize management strategy.

## Conclusion

Disease stage is the only prognostic factor for DFS, while age is the only independent risk factor for OS. Patients with metastatic MSO have an excellent disease-specific OS rate, regardless of surgical strategy, and a total thyroidectomy followed by radioiodine therapy should be advocated for in adjuvant therapy. We recommend conservative surgery in combination with total thyroidectomy followed by postoperative radioiodine therapy as the preferred method of disease management to achieve a favorable long-term quality of life in this population.

## Data Availability Statement

All datasets presented in this study are included in the article/Supplementary Material.

## Ethics Statement

The studies involving human participants were reviewed and approved by The Ethics Committee of Peking Union Medical College Hospital. The patients/participants provided their written informed consent to participate in this study. Written informed consent was obtained from the individual(s) for the publication of any potentially identifiable images or data included in this article.

## Author Contributions

SL and TY are the co-first authors who wrote the manuscript and participated in conceiving the study. LZ collected clinical data and completed the work of follow-up. XL conceived and designed the study. NC, HS, and JL had completed the surgery. All authors read and approved the manuscript.

## Conflict of Interest

The authors declare that the research was conducted in the absence of any commercial or financial relationships that could be construed as a potential conflict of interest.
