# Supplementary material for: FIGO Stage IV and Age Over 55 Years as Prognostic Predicators in Patients With Metastatic Malignant Struma Ovarii
Source: Front Oncol. 2020 Sep 29;10:584917. doi: 10.3389/fonc.2020.584917 (PMC7550973; doi:10.3389/fonc.2020.584917)
Supplement: Supplementary file 1 [file Table_1.DOCX]

Table S1. Database of our study.

| Reference | No. | Age(y) | pathology | Initial pathology | Time to  metastasis | Metastasis site | Surgery at metastasis | Adjuvant therapy | Results of follow-up |
| --- | --- | --- | --- | --- | --- | --- | --- | --- | --- |
|  | 1 | 39 | FTC | MSO | 9y | Lung | N | TT, RAI | AWD at 10y |
|  | 2 | 38 | FTC | Benign SO | 6y | Uterus, omentum, liver, lung | debulking surgery | TT, RAI | AWD at 44m |
|  | 3 | 33 | DTC | Benign SO | 5y | Pelvic bone  (Left acetabulum), peritoneum | Left ovarian cystectomy, metastases resection, bone biopsy | N | AWD at 15m |
|  | 4 | 34 | DTC | MSO | 2y | Peritoneum, vaginal residue | debulking surgery | Chemotherapy | AWD at 61m |
| Wynne  (1940) | 5 | 26 | FTC | Benign SO | 0.9y | Pelvic bone  ( Hip) | Metastases resection | N | Lack information |
| Woodruff &  Markley  (1957) | 6 | 26 | FTC | MSO | 5y | Lungs,  Anterior superior mediastinum | Metastases resection | RAI | AWD at 3y |
| Woodruff  (1966) | 7 | 52 | FTC | MSO | Initial | liver | Oophorectomy | radiotherapy | AWD at 7y |
| Kempers  (1970) | 8 | 58 | FTC | MSO | 16y | Peritoneum; lungs | debulking surgery | TT, RAI | NED at 5y |
|  | 9 | 46 | FTC | MSO | 7y | Liver, bone | TAH + BSO | N | DOD at 7.5y |
|  | 10 | 59 | FTC | MSO | Initial | Pelvic, lung | TAH + BSO | RAI | NED at 8y |
| Hasleton  (1978) | 11 | 73 | FTC | MSO(FTC) | Initial | Ileum, liver, omentum | NA | N | DOD at 2w later |
| Pardo-Mindan  Vasquez  (1983) | 12 | 60 | Mxed PTC + FTC | MSO  (FTC+PTC) | initial | Peritoneum, omentum;  Lungs, skin; lymph nodes | TAH + BSO | Chemotherapy, TT, EBRT | DOD at 2.5y |
| Willemse  (1987) | 13 | 36 | FTC | MSO | Initial | Contralateral ovary;  Omentum;  peritoneum | BSO | TT, RAI | NED at 0.6y |
| Rosenblum  (1989) | 14 | 37 | PTC | MSO (PTC) | Initial | Contralateral ovary;  peritoneum | NA | chemotherapy | AWD at 4.5y |
| Mcdougall  (1989) | 15 | 42 | FTC | Benign SO | 1.3y  (16m) | Bone (Throcic spine) | Metastatic lesion resection | TT, RAI | NED at 2y |
| O’Connell  (1990) | 16 | 35 | Mixed FTC+PTC | MSO(PTC) | 11y | Contralateral ovary; peritoneum | Debulking surgery | RAI | NED at 27y |
| Tokuda  (1993) | 17 | 28 | FTC | Benign SO | 3y | Bone (Cranial vault) | Metastatic lesion resection | N | Lack information |
| Brenner  (1996) | 18 | 49 | FTC | FTC | 0.8y | Contralateral ovary, Bladder | N | TT, RAI | NED at 2.5y |
| Vadmal  (1997) | 19 | 48 | FVPTC | Benign SO | 6y | Peritoneum,  Spleen, liver,  Diaphragmatic;  mesentery | Metastases resection | TT, RAI | NED at 1y |
| Tennvall  (1997) | 20 | 50 | FTC | FTC | Initial | Omentum;  Bladder;  Peritoneum  (pouch of  Douglas) | Debulking surgery | TT, RAI | NED at 6y |
| Mango  (1997) | 21 | 47 | MSO  (TC) | MSO | 0.5y | Pelvic bone (Ilium sacrum) | TAH + BSO | TT, RAI | AWD |
| Dardik  (1999) | 22 | 28 | FVPTC | MSO | 2y | Contralateral  fallopian tube;  paraaortic lymph node | debulking surgery | TT, RAI | NED at 2y |
|  | 23 | 39 | FTC; PTC in thyroid | MSO | 0.9y | Hernia sac;  Peritoneum;  Omentum. | debulking surgery | TT, RAI, EBRT | NED at 5y |
| Konez  (2000) | 24 | 46 | FTC | MSO | initial | Multiple liver metastases | NA | TT, RAI | Lack information |
| Rotman-  Pikielny  (2000) | 25 | 46 | FTC | FTC | Initial | Extensive liver metastasis | Ovarian cystectomy | TT, RAI, rhTSH | AWD at 0.5y |
| Chan  (2001) | 26 | 27 | FTC | Benign SO | 8m | Bone (Thoracic spine) | N | TT, RAI | AWD at 15m |
| Kouraklis  (2001) | 27 | 52 | TC | MSO | Initial | Ileum;  Retroperitoneum;  pelvic lymph nodes | Debulking surgery | TT, RAI | NED at 2y |
| Checrallah  (2001) | 28 | 42 | TC | Benign SO | 4y | Pelvic bones and lung | N | TT, RAI, rhTSH | AWD at 2y |
| Cherng  (2005) | 29 | 49 | TC | MSO | initial | peritoneal implants, liver metastases | Debulking surgery | TT, RAI | Lack information |
| Zekri  (2006) | 30 | 36 | FTC | MSO | 10y | Bone (Skull),  lung | N | TT, RAI | AWD at 6y |
| Mcdougall  (2006) | 31 | 58 | TC | MSO | initial | Liver, Bone  (lumbar spine) | N | TT, RAI | AWD at 6m |
| Schmidt  (2007) | 32 | 68 | PTC | Benign SO | 22y | Retroperitoneum | Debulking surgery | NA | AWD at 19y |
| Roth &  Karseladze  (2008) | 33 | 32 | FTC | Benign SO | 26y | Peritoneum; omentum; para-aortic lymph node | Debulking surgery | TT, RAI | AWD at 8y |
|  | 34 | 49 | FTC | FTC | Initial | omentum | TAH+BSO | Chemotherapy | NED at 16y+4m |
|  | 35 | 50 | FTC | Benign SO | Initial | omentum | Debulking surgery | TT, RAI | NED at 6y |
| Roth  (2008) | 36 | 26 | PTC | MSO | Initial | paraaortic lymph nodes | Ovarian cystectomy;  metastases resection | radiotherapy | NED at 6y |
|  | 37 | 70 | PTC  (poor differentiated) | Poorly differ  PTC | Initial | Uterus;  Peritoneum | TAH + BSO | TT, RAI  chemotherapy | DOD at 3y |
| Salvatori  (2008)  Fabbri  (2018) | 38 | 22  32 | FVPTC | Benign SO | 8y | peritoneum, diaphragm, vesical plica and liver | (Fertility-preserving)  conservative surgery | TT, RAI, rh-TSH | NED at 10y,  Transplantation of  cryopreserved ovarian failed |
| Janszen  (2008) | 39 | 36 | PTC | FVPTC | 3y | Liver, lymph nodes | N | TT, RAI | AWD at 4y |
| McGill  (2009) | 40 | 43 | FTC | MSO | 9y | Pelvic bone (Bilateral hips);  mediastinum | N | TT, RAI | AWD at 7y |
| Garg  (2009) | 41 | 38 | PTC | FVPTC | 3y | Contralateral ovary,  Peritoneum (cul-de-sac);  omentum | USO, metastases  resection, biopsies of left ovary, omentum, and peritoneum | TT, RAI | AWD at 6y |
|  | 42 | 34 | FVPTC | PTC; | 4y | Uterine serosa, pelvic sidewall, cul-de-sac,  diaphragm, liver | Debulking surgery | TT, RAI | AWD at 6y |
| Yamashita  (2010) | 43 | 32 | TC | Benign SO | 10m | Bone  (Lumbar spine) | Metastatic lesion resection | N | NED at 3y |
| Wolff  (2010) | 44 | 33 | FVPTC | Benign SO | 2y | Peritoneum, ometnum, diaphragm, cecal and jejunal serosal nodules, spleen | Debulking surgery | TT, RAI | AWD at 0.5y |
| Yang  (2010) | 45 | 76 | PTC | PTC | 31m | colon | Metastases resection | TT, RAI | NED at 1y |
| Sibio  (2010) | 46 | 74 | PTC | Brenner tumor  With PTC | Initial | peritoneum | Debulking surgery | N | NED at 7y |
| Ruel  (2010) | 47 | 42 | PTC | FVPTC | 15y | Lung, bone (rib); para-aortic lymph nodes | N | TT; RAI | NED at 0.5y |
| Marcy  (2010) | 48 | 45 | FVPTC | FVPTC | 1y | hypervascular liver metastases, peritoneunm;  adrenal; lung; Bone (ilium) | Debulking surgery | TT; RAI;  Chemotherapy | DOD at 3y |
| Minambres  (2011) | 49 | 38 | FTC | Benign SO | 13y | Pelvic bone  (Right iliac bone), lung, lymph nodes | BSO | TT, RAI | Died of respiratory failure at 14y |
| Lee  (2012) | 50 | 35 | FTC | FTC | 2y | Liver, diaphragm,  Right saplinx and  peritoneum, mesentery | Debulking surgery | TT, RAI | AWD at 0.5y |
| Steinman  (2013) | 51 | 22 | PTC | MSO | initial | Left anterior pelvis bone | USO | TT, RAI | NED at 5m |
|  | 52 | 49 | FVPTC | MSO | 11y | Pelvic bone  (Left hip) | Metastases resection | TT, RAI | NED at 4y |
| Collins  (2013) | 53 | 64 | PTC | PTC | 30m | peritoneum | Metastasis resection | TT, RAI | NED at 2.5y |
| Koo  (2014) | 54 | 41 | FTC | MSO | Initial | Peritoneum; spleen | Metastases resection | TT, RAI | Lack information |
| Ukita M  (2014) | 55 | 45 | FTC | MSO | Initial | Lung, bone (rib, right scapula, left  Acetabulum) | USO | TT, chemotherapy | AWD at 24y |
| Carey  (2014) | 56 | 70 | FTC;  PTC in thyroid | NA | initial | Peritoneum,  mediastinum | Debulking surgery | TT, RAI | AWD at 4m |
| Yan  (2014) | 57 | 38 | PTC | MSO | 2y | Liver; retroperitoneal lymph nodes | Metastases resection | TT, RAI, rh-TSH | AWD at 4y |
| Kim  (2014) | 58 | 38 | Anaplastic  TC | Benign  teratoma | Initial | Omentum;  Colon;  uterine serosa | Debulking surgery | TT, RAI | AWD |
| Luo  (2014) | 59 | 46 | PTC | MSO | Initial | Contralateral ovary, uterus, omentum, peritoneum | debulking surgery | TT, RAI | NED at 1y |
| Cong  (2015) | 60 | 38 | FTC | FTC | 17y | lung | N | TT, RAI | AWD at 3y |
| Kobayashi  (2015) | 61 | 49 | TC  (MSO) | Benign SO | 10y | Bone (Thoracic spine) | N | EBRT | NED at 9m |
| Seo  (2015) | 62 | 36 | FTC  (Poor differentiated) | FTC | Initial | Liver;  Peritoneum | Peritoneal and hepatic metastases resection | TT, RAI;  chemotherapy | NED at 4y |
| Wei  (2015) | 63 | 39 | FVPTC | FVPTC | initial | Round ligamenm;  Liver, peritoneum (cul-de-sac) | NA | TT, RAI | AWD at 10y |
| Riaz  (2015) | 64 | 21 | FVPTC | FVPTC | Initial | Omentum,  diaphragm, peritoneum, bone (skull, bilateral forearms), Lung | USO, omental  Biopsy | TT, RAI | AWD at 3m |
| Russo (2016) | 65 | 34 | FVPTC | Benign SO | 7y | Contralateral Ovary;  Appendix;  Peritoneum; omentum | Debulking, surgery | TT, RAI | NED at 8.6y |
| Zhu (2016) | 66 | 40 | PTC | MSO | Initial | omentum | Debulking surgery | chemotherapy | NED at 1y |
| Anagnostou  (2016) | 67 | 64 | FTC | FTC | initial | Peritoneum  (the pouch of Douglas) | Debulking surgery | N | NED at 4y |
| Williams  (2016) | 68 | 61 | PTC mixed  With  Insular  carcinoma | 90% insular carcinoma  with PTC | initial | uterine serosa | Debulking surgery | TT | NED at 3m |
| Oudoux  (2016) | 69 | 67 | PTC | PTC | initial | retrocaval lymph node, omentum | Debulking surgery | TT, RAI | NED at 5y |
| Comunello  (2017) | 70 | 38 | FTC | MSO | Initial | Bone  (Left scapula) | USO, partial scapulectomy | TT, RAI | NED at 1y |
| Gobitti  (2017) | 71 | 36 | FVPTC | FVPTC | Initial | Peritoneum;  Liver; uterus | Debulking surgery | TT, RAI, rh-TSH | AWD at 3m |
| Hassan  (2018) | 72 | 42 | PTC | PTC | Initial | Contralateral ovary | Debulking surgery | NA | Lack information;  TT, RAI proposed |
| Wu  (2018) | 73 | 48 | PTC | MSO | 14y | colon, rectum, liver, spleen, peritoneum | N | TT, RAI | AWD at 3m |
| Ernaga  (2018) | 74 | 57 | TC  (Poor differentiated) | FVPTC | Initial | Contralateral ovary; Liver;  Peritoneum | BSO | TT, RAI  Chemotherapy | DOD at 18m |
|  | 75 | 48 | FVPTC | FVPTC | Initial | Peritoneum | Debulking surgery | TT, RAI | NED at 3y |
| Lager (2018) | 76 | 30 | PTC | MSO | Initial | Bone (Rib;  proximal femurs;  sacrum); lung | USO | TT, RAI | AWD at 9m |
| Seifert (2019) | 77 | 67 | FVPTC | MSO | Initial | Bone (Femoral shaft) | BSO | RAI | NED at 30m |
| Szczepanek  (2019) | 78 | 19 | FVPTC | NA | 2m | Pelvic bone  (Left ilium) | USO | TT, RAI | NED at 9y |
| Gild  (2020) | 79 | 33 | FTC | Benign SO | 5y | Bone  (Scapula, Rib) | Scapular metastasis resection | TT, RAI | NED at 2y |

Abbreviations: SO, struma ovarii; MSO, malignant struma ovarii; PTC, papillary thyroid carcinoma; FTC, follicular thyroid carcinoma; FVPTC, follicular variant of papillary thyroid carcinoma; (D)TC, (differentiated) thyroid carcinoma; USO, unilateral salpingo-oophorectomy; BSO, bilateral salpingo-oophorectomy; TAH/sTAH, total/subtotal abdominal hysterectomy; TT, total thyroidectomy; RAI, radioiodine therapy; EBRT, external beam radiotherapy; rh-TSH, recombinant human thyroid stimulating hormone; NED, no evidence of disease; AWD, alive with disease; DOD, die of the disease; NA, not applicable.
