# Supplementary material for: FIGO Stage IV and Age Over 55 Years as Prognostic Predicators in Patients With Metastatic Malignant Struma Ovarii
Source: Front Oncol. 2020 Sep 29;10:584917. doi: 10.3389/fonc.2020.584917 (PMC7550973; doi:10.3389/fonc.2020.584917)
Supplement: Supplementary file 2 [file Table_2.DOCX]

**Table S2** Univariate and multivariate analysis of clinical outcomes

| Factors | NED | AWD/DOD | t/X^2^ | P value | Logistic regression analysis |
| --- | --- | --- | --- | --- | --- |
|  |  |  |  |  | OR (95%CI) P |
| Age (y) | 45.8±14.8 | 42.8±12.7 | 0.943 | 0.349 |  |
| Metastatic disease at initial presentation, n (%) |  |  | 1.051 | 0.305 |  |
| Yes | 18(51.4%) | 15(39.5%) |  |  |  |
| No | 17(48.6%) | 23(60.5%) |  |  |  |
| FIGO Stage, n (%) ^a^ |  |  | 10.037 | 0.002* | 5.333 (1.839, 15.471) 0.002* |
| II-III | 20(57.1%） | 8(21.1%) |  |  |  |
| IV | 15(42.9%) | 30(78.9%) |  |  |  |
| Follicular carcinoma subtype, n (%) |  |  | 0.765 | 0.382 |  |
| Yes | 13(40.6%) | 16(51.6%) |  |  |  |
| No | 19(59.4%) | 15(48.4%) |  |  |  |
| Surgical options, n (%) ^a^ |  |  | 3.654 | 0.161 |  |
| No surgery | 3(8.6%) | 9(25.7%) |  |  |  |
| Conservative surgery | 14(40.0%) | 12(34.3%) |  |  |  |
| Aggressive surgery | 18(41.4%) | 14(40%) |  |  |  |
| RAI therapy, n (%) |  |  | 0.035 | 0.852 |  |
| Yes | 27(77.1%) | 30(21.1%) |  |  |  |
| No | 8(22.9%) | 8(78.9%) |  |  |  |

Abbreviations: RAI, radioiodine therapy; NED, no evidence of disease; AWD, alive with disease; DOD, die of the disease.

*a* Factors applied to multivariate analysis; * *p* < 0.05*
