# Supplementary material for: FIGO Stage IV and Age Over 55 Years as Prognostic Predicators in Patients With Metastatic Malignant Struma Ovarii
Source: Front Oncol. 2020 Sep 29;10:584917. doi: 10.3389/fonc.2020.584917 (PMC7550973; doi:10.3389/fonc.2020.584917)
Supplement: Supplementary file 3 [file Table_3.DOCX]

**Table S3** Univariate and multivariate analysis of OS

| Factors | N |  | Univariate analysis | | |  | Multivariate cox regression analysis | | | |
| --- | --- | --- | --- | --- | --- | --- | --- | --- | --- | --- |
|  |  | Mean survival(yrs) | | 10-year survival rate | p |  | OR | (95% CI) | | p |
| Age (<55/>=55, years) ^a^ | 55/16 | 21.7/12.6 | | 88.1%/62.5% | 0.009* |  | 9.362 | | 1.895, 46.246 | 0.006* |
| Metastatic disease at initial presentation (Yes/No) | 32/39 | 19.5/20.2 | | 79.6%/85.2% | 0.290 |  |  | | | |
| FIGO Stage (II-III /IV) ^a^ | 26/43 | 25.6/15.3 | | 94.1%/75.0% | 0.098 |  |  |  | |  |
| Follicular carcinoma subtype (Yes/No) | 29/32 | 23.3/18.1 | | 84.7%/82.8% | 0.952 |  |  | | | |
| Surgical options |  |  |  | | |  |  | | | |
| No surgery/Conservative surgery | 12/26 | - | | -/94.1% | 0.521 |  |  | | | |
| No surgery/Aggressive surgery | 12/30 | - | | -/94.1% | 0.320 |  |  | | | |
| Conservative surgery/Aggressive surgery | 26/30 | - | | 94.1%/72.9% | 0.746 |  |  | | | |
| RAI therapy (Yes/No) | 55/16 | 18.8/17.0 | | 90.7%/63.3% | 0.411 |  |  | | | |

Abbreviations: RAI, radioiodine therapy; NED, no evidence of disease; AWD, alive with disease; DOD, die of the disease.

*a* Factors applied to multivariate analysis; * *p* < 0.05*
