# Supplementary figures and images for: FIGO Stage IV and Age Over 55 Years as Prognostic Predicators in Patients With Metastatic Malignant Struma Ovarii
Source: Front Oncol. 2020 Sep 29;10:584917. doi: 10.3389/fonc.2020.584917 (PMC7550973; doi:10.3389/fonc.2020.584917)

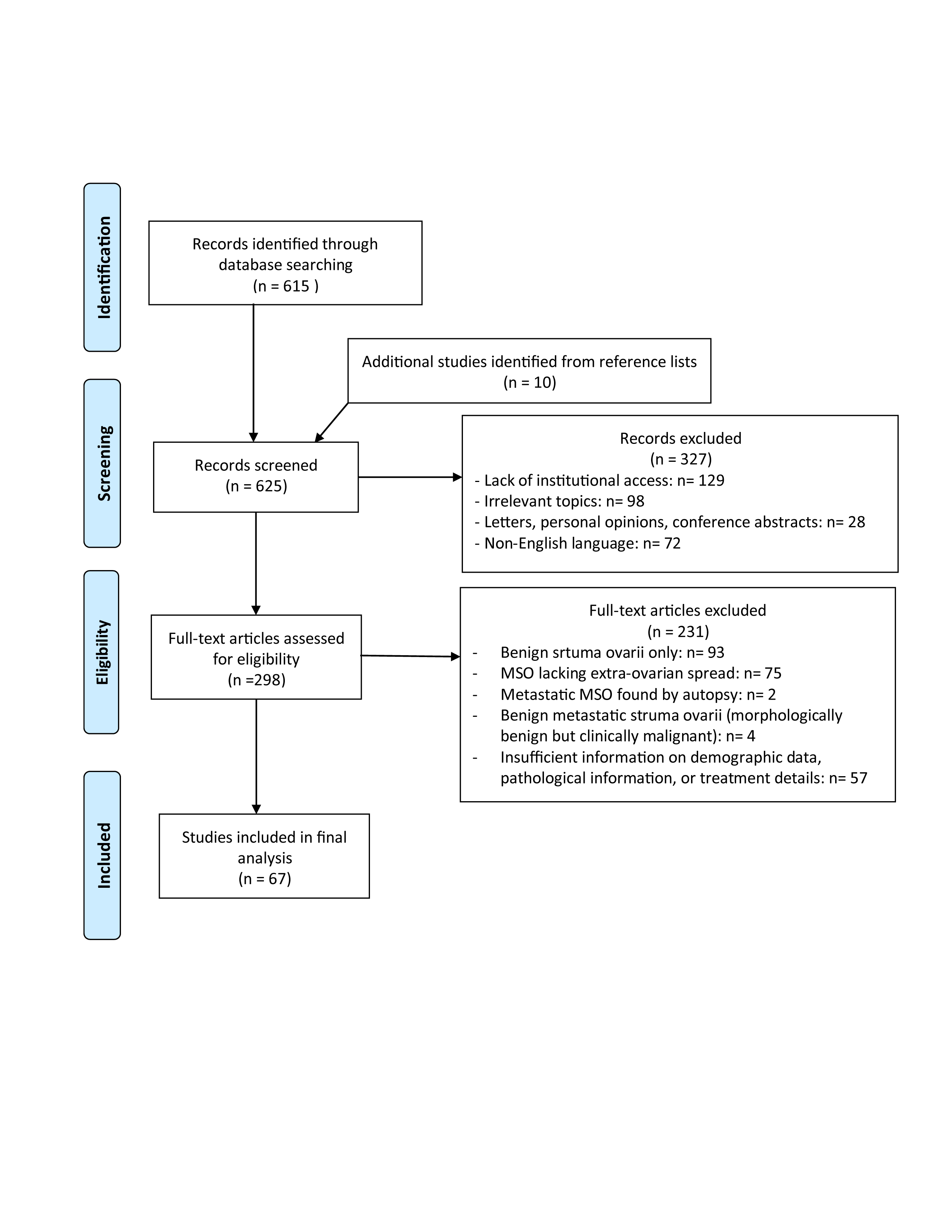

Supplement: Supplementary file 4 [file Image_1.tif]
